# Association of Adult Weight Gain With Major Health Outcomes Among Middle-aged Chinese Persons With Low Body Weight in Early Adulthood

**DOI:** 10.1001/jamanetworkopen.2019.17371

**Published:** 2019-12-13

**Authors:** Guochong Jia, Xiao-Ou Shu, Ying Liu, Hong-Lan Li, Hui Cai, Jing Gao, Yu-Tang Gao, Wanqing Wen, Yong-Bing Xiang, Wei Zheng

**Affiliations:** 1 Vanderbilt Epidemiology Center, Division of Epidemiology, Department of Medicine, Vanderbilt-Ingram Cancer Center, Vanderbilt University Medical Center, Nashville, Tennessee; 2 Department of Epidemiology, Shanghai Cancer Institute, Shanghai, People’s Republic of China

## Abstract

**Importance:**

The association of weight gain from early to middle adulthood with disease risk has not been adequately studied.

**Objective:**

To investigate the association of adult weight gain with major health outcomes in a Chinese population with low body weight in early adulthood.

**Design, Setting, and Participants:**

This population-based cohort study assessed data from 48 377 women and 35 989 men aged 40 to 59 years at recruitment in 2 prospective cohort studies in China. The Shanghai Women’s Health Study recruited 74 941 women, aged 40 to 70 years, from January 1, 1996, to December 31, 2000, and the Shanghai Men’s Health Study recruited 61 482 men, aged 40 to 74 years, from January 1, 2002, to December 31, 2006. This analysis was conducted from September 1, 2017, to April 30, 2018.

**Exposures:**

Weight gain from 20 years of age to 40 to 59 years of age.

**Main Outcomes and Measures:**

Mortality and incidence of cancers and other chronic diseases.

**Results:**

This analysis included 48 377 women (mean [SD] age, 47.8 [5.3] years) and 35 989 men (mean [SD] age, 49.6 [5.1] years). Per 5-kg weight gain from early to middle adulthood was associated with an approximately 10% (hazard ratio [HR], 1.09; 95% CI, 1.04-1.14 for men; HR, 1.14; 95% CI, 1.11-1.19 for women) elevated all-cause mortality and a greater than 20% (HR, 1.26; 95% CI, 1.16-1.38 for men; HR, 1.23; 95% CI, 1.14-1.33 for women) cardiovascular disease–related mortality in later life among individuals who reached a body mass index (BMI) of 23 or higher at middle adulthood. Body mass index at middle adulthood also modified the association of weight gain with risk of obesity-related cancers, with weight gain of 20 kg or more associated with increased risks both for men (HR, 1.34; 95% CI, 1.07-1.67) and for women (HR 1.45; 95% CI, 1.24-1.68). No similar associations were found for individuals with a BMI of 18.5 to 22.9. Regardless of BMI, weight gain was associated with elevated risks of type 2 diabetes, hypertension, fatty liver disease, stroke, gout, and gallstones, particularly for type 2 diabetes (HR, 7.87; 95% CI, 6.91-8.97 for women; HR, 4.95; 95% CI, 4.23-5.79 for men) and fatty liver disease (HR, 3.68; 95% CI, 3.42-3.95 for women; HR, 2.83, 95% CI, 2.56-3.13 for men) in individuals with weight gain of 20 kg or more compared with those with a healthy weight.

**Conclusions and Relevance:**

This study found that weight gain from early to middle adulthood was associated with disease incidence and mortality in later life. The BMI at middle adulthood modified the association of weight gain with mortality and cancer incidence but not risk of other major chronic diseases.

## Key Points

**Question:**

What is the association between weight gain from early to middle adulthood and major health outcomes in later life given the body mass index at middle adulthood?

**Findings:**

In this population-based cohort study of 2 Chinese cohorts of 48 377 women and 35 989 men, weight gain from early to middle adulthood was associated with elevated total and major cause-specific mortality as well as incidence of multiple obesity-related cancers in later life only among those who reached a body mass index of 23 or higher at middle adulthood. Positive associations of weight gain with risk of type 2 diabetes, hypertension, fatty liver disease, stroke, gout, and gallstones were found regardless of body mass index level (≥23 or <23) in middle adulthood.

**Meaning:**

A moderate weight gain from early to middle adulthood may be associated with elevated risk of some chronic diseases in later life.

## Teaser

This population-based cohort study investigates the association of adult weight gain with major health outcomes in middle-aged Chinese persons who had low body weight in early adulthood.

## Introduction

Obesity, a condition related to excess adiposity, has been associated with increased risk of multiple chronic diseases, including type 2 diabetes, cardiovascular diseases, stroke, hypertension, fatty liver disease, and certain types of cancers.^1^ The epidemic of obesity has been a serious health concern in the United States and multiple high-income countries during the past 2 decades.^2,3^ In the past, most Asian individuals have had a low body weight.^4^ With rapid economic development and adoption of sedentary lifestyles during the past few decades, there has been a marked increase in obesity and obesity-related diseases in many Asian countries, such as China, the most populated country in the world.^2,3^ The number of obese adults in China has already surpassed that in the United States.^5^

Although obesity among children and adolescents has become increasingly common in the past few decades, most middle-aged or elderly individuals with obesity cumulate their excessive adiposity by gaining weight from early to middle adulthood.^6^ However, most previous studies^7,8^ on excess adiposity investigated only the body mass index (BMI) (calculated as weight in kilograms divided by height in meters squared) at middle adulthood and its association with health outcomes in later life. Some previous studies^9,10^ have investigated weight gain in adulthood in relation to a wide range of health outcomes. Few of them, however, have defined weight gain across specific age ranges or systematically quantified the association of weight gain from early to middle adulthood with risk of diseases and death. A recent study^11^ conducted in the United States found that even a moderate weight gain from early to middle adulthood (<10.0 kg) was associated with increased total mortality and risk of major chronic diseases, such as type 2 diabetes, cardiovascular disease, hypertension, and obesity-related cancers. It is unclear to what extent these findings could be extrapolated to Asian individuals. Given that many Asian individuals have a low body weight in early adulthood, a moderate weight gain from early to middle adulthood may not lead to being overweight at middle adulthood, and it may not be necessarily detrimental. However, Asian individuals have a higher risk of type 2 diabetes, hypertension, and hyperlipidemia than other populations at a relatively low BMI^12,13,14^; thus, it is possible that a moderate weight gain may have stronger associations with health outcomes in Asian populations than in those of European descent. In the present study, using data from 2 large population-based cohort studies, we evaluated the association of weight gain from early to middle adulthood with total and cause-specific mortality, the risk of cancers, and risk of other major chronic diseases in later life in a Chinese population.

## Methods

### Study Population

This cohort study used data from the Shanghai Women’s Health Study (SWHS) and the Shanghai Men’s Health Study (SMHS), which are 2 ongoing population-based prospective cohort studies. The SWHS recruited 74 941 women aged 40 to 70 years from January 1, 1996, to December 31, 2000, and the SMHS recruited 61 482 men aged 40 to 74 years from January 1, 2002, to December 31, 2006. Details of the study designs and methods have been published elsewhere.^15,16^ In brief, information on sociodemographic characteristics, anthropometric characteristics, usual dietary intakes, lifestyle factors, and medical history was collected via in-person interviews at baseline. Participants of both cohorts were followed up for cause-specific mortality and incidence of site-specific cancers and other major chronic diseases through a combination of linkages to the Shanghai Vital Statistics and Cancer Registries and in-person surveys conducted every 2 to 4 years. Both the SWHS and SMHS have been approved by the institutional review boards of the Shanghai Cancer Institute and the Vanderbilt University Medical Center, and all participants provided written informed consent. All data were deidentified. This study followed the Strengthening the Reporting of Observational Studies in Epidemiology (STROBE) reporting guideline.

### Assessment of Weight Gain

At the baseline interview, study participants were asked to recall their weight in kilograms at 20 years of age, and their current weight and height were measured.^17,18^ Participants who were between 40 and 59 years of age at the time of the baseline survey were considered to be at middle adulthood and thus included in the current analysis. The recalled weight at 20 years of age was used as the early adulthood measure. Weight change from early to middle adulthood was computed by subtracting the recalled weight at 20 years of age from measured weight at baseline.

### Outcomes Ascertainment

Cancer cases and deaths were identified via record linkage with the population-based cancer registry and data collected at the Vital Statistic Unit, followed by home visits or telephone calls if necessary to confirm the diagnoses. Cancer diagnoses were verified by a review of medical records obtained from the diagnosing hospital. Additional details regarding collection of outcome data have been described previously.^15,16^ The outcomes analyzed in the present study include deaths in the cohort follow-up through December 31, 2016 (median follow-up, 15.2 years for SWHS and 9.3 years for SMHS), and newly diagnosed cancer and other chronic diseases through December 31, 2014.

Causes of death and diagnoses of cancers were categorized according to the *International Classification of Diseases, Ninth Revision* (*ICD-9*).^19^ Outcomes in this study included deaths from all causes, cardiovascular diseases (*ICD-9* codes 390-459), cancers (*ICD-9* codes 140-208), and all other causes, as well as newly diagnosed cases of all cancers combined (*ICD-9* codes 140-208) and site-specific cancers known to be associated with obesity, including cancer of the colon and rectum (*ICD-9* codes 153-154), breast (*ICD-9* code 174), corpus uteri (*ICD-9* code 182), and ovary (*ICD-9* code 183). In addition to these cancers, previous studies^20,21^ also reported an association of obesity with cancer of the gastric cardia (*ICD-9* code 151.0), liver (*ICD-9* code 155), gallbladder (*ICD-9* code 156), pancreas (*ICD-9* code 157), kidney (*ICD-9* code 189), and thyroid (*ICD-9* codes 193) and multiple myeloma (*ICD-9* code 203.0); all these cancers were grouped together as obesity-related cancers in our data analyses. We also present results for lung cancer (*ICD-9* code 162) and noncardia gastric cancer (ICD-9 code 151.1-151.9) because they are also among common cancers diagnosed in these cohorts.

Other chronic diseases evaluated in this study were type 2 diabetes, hypertension, acute myocardial infarction, stroke (hemorrhagic or ischemic), fatty liver disease, gallstones, cholecystitis, and gout. Information of these outcomes was collected via in-person follow-up surveys. Individuals who had 1 of the following 4 criteria were considered to have type 2 diabetes: (1) fasting glucose levels of 126 mg/dL or higher on 2 separate occasions; (2) 2-hour postprandial glucose levels of 200 mg/dL or higher on 2 occasions; (3) use of hypoglycemic medication; or (4) presence of any diabetes symptoms (frequent urination, increased thirst, increased hunger, and unexplained weight loss) plus fasting glucose levels of 126 mg/dL or higher or 2-hour postprandial glucose levels of 200 mg/dL or higher (to convert glucose values to millimoles per liter, multiply by 0.0555).

### Statistical Analysis

Participants were excluded from all analyses if they were younger than 40.0 years or older than 59.9 years at recruitment; had missing data for recalled weight at 20 years of age, vital status, or height measurements; or had been diagnosed with cancer, stroke, or myocardial infarction at baseline (23 855 women and 23 531 men). Underweight participants (BMI <18.5 at baseline) were excluded because the sample size for this group was too small for any informative analyses (1745 women and 1668 men). We further excluded all female ever smokers (n = 740) given the small number of women in this group. We also excluded participants who died or were lost to follow-up within the first 3 years from study enrollment (224 women and 290 men). Data from 48 377 women and 35 989 men remained after these exclusions. In addition, participants with an in situ cancer diagnosis or an unconfirmed cancer diagnosis were excluded from analyses of cancers (242 women and 63 men). Women who reported a hysterectomy (n = 2234) were excluded from the analyses of cancer of corpus uteri.

Given that BMI at middle adulthood is associated with death and diseases in later life, analyses of adult weight gain were conducted by strata of BMI at study enrollment. Multiple studies^22,23^ have found that Asian individuals have a higher risk of type 2 diabetes, hypertension, and hyperlipidemia than European descendants at a relatively low BMI and proposed a BMI of 23 to define overweight in Asian individuals. We defined overweight as a BMI of 23 or higher in our analyses. Cox proportional hazards regression models stratified by birth cohort, with age as the time scale, were used to investigate the association of weight gain with total and cause-specific mortality, as well as the incidence of common cancers and other chronic diseases. A Fine-Gray model of competing risk was used for analyses of cause-specific mortality. Hazard ratios (HRs) and 95% CIs were estimated per 5-kg increment in weight gained. We also estimated HRs for participants who gained more than 20 kg from early to middle adulthood and reached a BMI of 23 or higher at middle adulthood compared with participants with a healthy weight at middle adulthood (BMI of 18.5-22.9). Participants who gained more than 50 kg (24 women and 18 men) were denoted as having a weight gain of 50 kg to minimize the effect of outliers. The follow-up time was calculated after the exclusion of the first 3 years of cohort observations.

We adjusted for the following covariates in multivariable analyses: age at study enrollment, recalled weight at 20 years of age, height, educational level (elementary or middle school, high school graduate, or some college or more), smoking status (men only: never smoker, past smoker, and current smoker), pack-years of smoking (men only: <5, 5-19, ≥20), regular alcohol consumption (yes or no), total physical activity level (quintiles), dietary patterns (healthy eating index^24^ in quintiles), family cancer history (yes or no), menopausal status (women only: premenopausal or postmenopausal), female hormone therapy (women only: ever or never), parity (women only: nulliparous, 1 child, or ≥2 children). The potential nonlinear association between weight gain and mortality was evaluated with restricted cubic spline function. We performed additional analyses stratified by smoking status (ever vs never) in men to evaluate the potential association with smoking. All tests were 2-sided at a significance level of α = .05. All statistical analyses were conducted using SAS, version 9.4 (SAS Institute Inc) and R, version 3.3.3 (R Project for Statistical Computing).

## Results

This analysis included 48 377 women (mean [SD] age, 47.8 [5.3] years) and 35 989 men (mean [SD] age, 49.6 [5.1] years). At baseline, women were more likely to have a BMI lower than 23, a lower educational level, and more physical activity than men (eTable 1 in the Supplement). The mean (SD) body weight at early adulthood was 49.7 (6.5) kg for women and 57.1 (6.4) kg for men. Men had a higher mean weight gain than women from early to middle adulthood. Only 6316 women (13.1%) and 7609 men (21.1%) gained 20 kg or more of weight during this period, and more than half of the participants had a BMI of 23 or higher at middle adulthood.

Among lifetime never-smoking women, elevated mortality from any cause, cardiovascular diseases (CVDs), and cancers were associated with weight gain from early to middle adulthood (Table 1). However, these associations were observed only among women who gained weight and reached a BMI of 23 or higher at middle adulthood (40-59 years of age). Elevated HRs were found for the associations with total mortality (1.14; 95% CI, 1.10-1.19) and CVD-related mortality (1.23; 95% CI, 1.14-1.33). Similarly, for men who gained weight and reached a BMI of 23 or higher at middle adulthood, elevated HRs were found for all-cause deaths (HR, 1.09; 95% CI, 1.04-1.14) and CVD-related deaths (HR, 1.26; 95% CI, 1.16-1.38) per 5-kg weight increase. However, for men with a healthy weight at middle adulthood, weight gain was associated with reduced mortality from all causes (HR, 0.83; 95% CI, 0.75-0.93) or cancers (HR, 0.81; 95% CI, 0.71-0.93) (Table 1), particularly among male smokers (eTable 2 in the Supplement). In general, the association between weight gain and mortality was linear among men and women who reached a BMI of 23 or higher at middle adulthood (eFigure 1 in the Supplement).

**Table 1.** Adjusted HRs for Total and Cause-Specific Mortality per 5-kg Weight Gain From Early to Middle Adulthood, Stratified by BMI at Middle Adulthood in the Shanghai Cohort Studies, 1996-2016

Mortality	Total			BMI						*P* Value for Interaction
				18.5-22.9			≥23			
	No. of Deaths (n = 48 377 Women and 35 989 Men)	HR (95% CI)^a^	*P* Value	No. of Deaths (n = 21 154 Women and 14 218 Men)	HR (95% CI)^a^	*P* Value	No. of Deaths (n = 27 223 Women and 21 771 Men)	HR (95% CI)^a^	*P* Value	
**Women**^**b**^										
All-cause	2171	1.10 (1.07-1.13)	<.001	768	0.97 (0.86-1.09)	.58	1403	1.14 (1.10-1.19)	<.001	.002
CVD	407	1.20 (1.13-1.28)	<.001	107	0.91 (0.65-1.27)	.56	300	1.23 (1.14-1.33)	<.001	.25
Cancer	1300	1.06 (1.02-1.10)	.004	497	0.94 (0.80-1.09)	.40	803	1.10 (1.04-1.16)	.001	.02
Other	464	1.10 (1.03-1.17)	.004	164	1.11 (0.86-1.43)	.44	300	1.15 (1.05-1.26)	.003	.08
**Men**										
All-cause	1644	1.00 (0.97-1.03)	.84	663	0.83 (0.75-0.93)	.001	981	1.09 (1.04-1.14)	.001	.08
CVD	399	1.14 (1.07-1.21)	<.001	142	0.94 (0.74-1.20)	.63	257	1.26 (1.16-1.38)	<.001	.08
Cancer	887	0.95 (0.91-1.00)	.04	376	0.81 (0.71-0.93)	.003	511	1.02 (0.95-1.10)	.59	.02
Other	358	0.95 (0.89-1.02)	.13	145	0.80 (0.64-1.01)	.06	213	1.01 (0.91-1.13)	.86	.04

Abbreviation: BMI, body mass index (calculated as weight in kilograms divided by square of height in meters); CVD, cardiovascular disease; HR, hazard ratio.

^a^ The HRs were estimated per 5-kg weight gain, adjusted for age at study enrollment, recalled weight at 20 years of age, height, educational level, smoking status, pack-years of smoking, regular alcohol consumption, total physical activity, healthy eating index, family cancer history, menopausal status (women only), female hormone replacement therapy (women only), parity (women only), and birth cohorts.

^b^ Female ever smokers were excluded from analyses.

Among women who reached a BMI of 23 or higher at middle adulthood, each 5-kg increase in weight was associated with an elevated risk of all cancers, obesity-related cancers, postmenopausal breast cancer, cancer of the corpus uteri, and liver cancer (Table 2). In particular, a greater than 2-fold increase in risk was observed for postmenopausal breast cancer (HR, 2.20; 95% CI, 1.51-3.22) and cancer of the corpus uteri (HR, 2.75; 95% CI, 1.83-4.13) in association with a weight gain of 20 kg or higher compared with women with healthy weight. Among men, similar associations were observed only for obesity-related cancer (HR, 1.34; 95% CI, 1.07-1.67), and a reduced risk of lung cancer was observed (HR, 0.58; 95% CI, 0.39-0.87).

**Table 2.** Adjusted HRs for Incidence of Cancers With Weight Gain from Early to Middle Adulthood in the Shanghai Cohort Studies, 1996-2014

Cancer Site	No. of Cases	Per 5-kg Weight Gain^a^		≥20-kg Weight Gain^b^	
		HR (95% CI)^c^	*P* Value	HR (95% CI)^c^	*P* Value
**Women**^**d**^					
All cancers	1829	1.06 (1.02-1.10)	.001	1.27 (1.14-1.42)	<.001
Obesity-related cancers^e^	966	1.09 (1.04-1.15)	.001	1.45 (1.24-1.68)	<.001
Postmenopausal breast cancer	190	1.15 (1.04-1.28)	.008	2.20 (1.51-3.22)	<.001
Corpus uteri	115	1.37 (1.22-1.55)	<.001	2.75 (1.83-4.13)	<.001
Colorectum	278	0.99 (0.90-1.09)	.81	1.19 (0.89-1.59)	.24
Liver	46	1.25 (1.02-1.52)	.03	1.79 (0.89-3.59)	.10
Thyroid	131	0.93 (0.79-1.09)	.35	1.25 (0.81-1.92)	.32
Premenopausal breast cancer	320	1.00 (0.91-1.10)	.97	0.96 (0.73-1.26)	.77
Stomach (noncardia only)	103	1.10 (0.94-1.27)	.24	0.92 (0.55-1.52)	.73
Lung	190	0.89 (0.78-1.02)	.09	0.89 (0.63-1.27)	.52
All other cancers	250	1.12 (1.02-1.23)	.02	1.63 (1.23-2.16)	.001
**Men**					
All cancers	817	1.02 (0.96-1.08)	.53	1.06 (0.91-1.23)	.49
Obesity-related cancers^e^	396	1.10 (1.01-1.18)	.02	1.34 (1.07-1.67)	.01
Colorectum	158	1.10 (0.97-1.25)	.12	1.39 (0.97-1.99)	.08
Liver	82	1.01 (0.85-1.21)	.91	0.91 (0.54-1.53)	.72
Thyroid	37	1.20 (0.93-1.53)	.16	1.92 (0.97-3.80)	.06
Stomach (noncardia only)	70	1.05 (0.87-1.26)	.63	0.89 (0.52-1.53)	.67
Lung	129	0.86 (0.73-1.01)	.07	0.58 (0.39-0.87)	.009
All other cancers	222	0.96 (0.86-1.08)	.49	1.07 (0.80-1.42)	.65

Abbreviation: HR, hazard ratio.

^a^ The HRs were estimated per 5-kg weight gain among individuals who had a body mass index (BMI) (calculated as weight in kilograms divided by square of height in meters) of 23 or higher at middle adulthood.

^b^ The HRs were estimated for individuals who had a weight gain of 20 kg or more and reached a BMI of 23 or higher at middle adulthood compared with those with a healthy weight at middle adulthood (BMI, 18.5-22.9).

^c^ The HRs were adjusted for age at study enrollment, recalled weight at 20 years of age, height, educational level, smoking status, pack-years of smoking, regular alcohol consumption, total physical activity, healthy eating index, family cancer history, menopausal status (women only), female hormone replacement therapy (women only), parity (women only), and birth cohorts.

^d^ Female ever smokers were excluded from analyses.

^e^ Obesity-related cancers included cancers of gastric cardia, colon or rectum, liver, gallbladder, pancreas, renal cell, thyroid, and multiple myeloma.

Among those who reached a BMI of 23 or higher at middle adulthood, increased risks were observed for multiple other chronic diseases (Table 3). Among women, a weight gain of 20 kg or higher was associated with an elevated risk of all chronic diseases evaluated in Table 3. The positive association may not be linear for acute myocardial infarction and cholecystitis because the association was not statistically significant per 5-kg weight gain. The association was strongest for type 2 diabetes (HR, 7.87; 95% CI, 6.91-8.97), followed by gout (HR, 3.93; 95% CI, 3.07-5.03) and fatty liver disease (HR, 3.68; 95% CI, 3.42-3.95). Similar positive associations were observed for men.

**Table 3.** Adjusted HRs for Incidence of Other Chronic Diseases With Weight Gain from Early to Middle Adulthood in the Shanghai Cohort Studies, 1996-2014

Disease	No. of Cases	Per 5-kg Weight Gain^a^		≥20-kg Weight Gain^b^	
		HR (95% CI)^c^	*P* Value	HR (95% CI)^c^	*P* Value
**Women**^**d**^					
Type 2 diabetes	1995	1.45 (1.41-1.49)	<.001	7.87 (6.91-8.97)	<.001
Hypertension	3397	1.13 (1.10-1.16)	<.001	1.87 (1.72-2.04)	<.001
Acute myocardial infarction	214	1.06 (0.96-1.18)	.26	2.10 (1.47-3.02)	<.001
Stroke	2316	1.11 (1.07-1.14)	<.001	1.57 (1.41-1.74)	<.001
Hemorrhagic stroke	91	1.31 (1.14-1.50)	<.001	2.99 (1.80-4.97)	<.001
Ischemic stroke	2203	1.10 (1.06-1.13)	<.001	1.53 (1.38-1.70)	<.001
Fatty liver	5371	1.24 (1.22-1.27)	<.001	3.68 (3.42-3.95)	<.001
Gallstones	1686	1.11 (1.07-1.15)	<.001	1.93 (1.71-2.18)	<.001
Cholecystitis	527	1.05 (0.98-1.13)	.16	1.36 (1.10-1.68)	.005
Gout	483	1.22 (1.14-1.30)	<.001	3.93 (3.07-5.03)	<.001
**Men**					
Type 2 diabetes	1140	1.38 (1.33-1.44)	<.001	4.95 (4.23-5.79)	<.001
Hypertension	1487	1.10 (1.05-1.15)	<.001	1.62 (1.45-1.82)	<.001
Acute myocardial infarction	235	1.17 (1.06-1.29)	.001	1.86 (1.41-2.45)	<.001
Stroke	874	1.11 (1.05-1.17)	<.001	1.58 (1.35-1.84)	<.001
Hemorrhagic stroke	59	1.27 (1.06-1.53)	.009	1.50 (0.85-2.65)	.17
Ischemic stroke	803	1.09 (1.04-1.16)	.001	1.56 (1.33-1.83)	<.001
Fatty liver	2301	1.18 (1.14-1.22)	<.001	2.83 (2.56-3.13)	<.001
Gallstones	472	1.12 (1.04-1.20)	.003	1.58 (1.28-1.94)	<.001
Cholecystitis	224	1.03 (0.93-1.16)	.56	1.52 (1.12-2.07)	.008
Gout	424	1.19 (1.11-1.28)	<.001	2.46 (1.95-3.12)	<.001

Abbreviation: HR, hazard ratio.

^a^ The HRs were estimated per 5-kg weight gain among individuals who had a body mass index (BMI) (calculated as weight in kilograms divided by square of height in meters) of 23 or higher at middle adulthood.

^b^ The HRs were estimated for individuals who had a weight gain of 20 kg or more and reached a BMI of 23 or higher at middle adulthood compared with those with a healthy weight at middle adulthood (BMI, 18.5-22.9).

^c^ The HRs were adjusted for age at study enrollment, recalled weight at 20 years of age, height, educational level, smoking status, pack-years of smoking, regular alcohol consumption, total physical activity, healthy eating index, family cancer history, menopausal status (women only), female hormone replacement therapy (women only), parity (women only), and birth cohorts.

^d^ Female ever smokers were excluded from analyses.

Table 4 details the association of weight gain with selected diseases among individuals who had a BMI of 18.5 to 22.9 at middle adulthood. Except for an inverse association with lung cancer for men, no significant associations between weight gain and risks of cancers were observed. However, weight gain was associated with elevated risks of type 2 diabetes for both women and men (HR, 1.71; 95% CI, 1.42-2.06 for women; HR, 1.85; 95% CI, 1.51-2.26 for men), hypertension (HR, 1.32; 95% CI, 1.23-1.43 for women; HR, 1.28; 95% CI, 1.16-1.41 for men), ischemic stroke (HR, 1.11; 95% CI, 1.00-1.24 for women; HR, 1.28; 95% CI, 1.10-1.49 for men), fatty liver disease (HR, 2.01; 95% CI, 1.85-2.19 for women; HR, 1.69; 95% CI, 1.52-1.89 for men), and gallstones (HR, 1.27; 95% CI, 1.13-1.42 for women; HR, 1.25; 95% CI, 1.03-1.53 for men). In addition, among men, weight gain was also associated with risk of acute myocardial infarction (HR, 1.43; 95% CI, 1.07-1.89), cholecystitis (HR, 1.58; 95% CI, 1.16-2.16), and gout (HR, 1.35; 95% CI, 1.05-1.73).

**Table 4.** Adjusted HRs for Incidence of Chronic Diseases per 5-kg Weight Gain from Early to Middle Adulthood Among Individuals With Healthy Weight at Middle Adulthood^a^

Disease	Women^b^			Men		
	No. of Cases	HR (95% CI)^c^	*P* Value	No. of Cases	HR (95% CI)^c^	*P* Value
All cancers	1203	1.01 (0.92-1.11)	.87	485	0.97 (0.85-1.09)	.59
Obesity-related cancers^d^	554	1.10 (0.95-1.26)	.20	195	1.14 (0.93-1.39)	.22
Postmenopausal breast cancer	61	0.84 (0.56-1.28)	.43	NA	NA	NA
Endometrium cancer	55	0.90 (0.58-1.38)	.63	NA	NA	NA
Colorectal	176	1.11 (0.86-1.43)	.43	74	1.14 (0.82-1.59)	.43
Liver	23	0.88 (0.45-1.73)	.71	46	0.93 (0.62-1.38)	.71
Thyroid	94	1.25 (0.88-1.76)	.21	17	1.00 (0.51-1.95)	.99
Premenopausal breast cancer	284	1.11 (0.91-1.35)	.32	NA	NA	NA
Stomach (noncardia only)	69	1.02 (0.68-1.53)	.92	46	1.05 (0.69-1.58)	.84
Lung	141	0.78 (0.59-1.02)	.07	106	0.77 (0.59-0.99)	.05
All other cancers	155	0.82 (0.63-1.06)	.12	138	0.91 (0.72-1.15)	.41
Type 2 diabetes	360	1.71 (1.42-2.06)	<.001	236	1.85 (1.51-2.26)	<.001
Hypertension	2002	1.32 (1.23-1.43)	<.001	849	1.28 (1.16-1.41)	<.001
Acute myocardial infarction	76	1.00 (0.68-1.48)	.99	107	1.43 (1.07-1.89)	.01
Stroke	1077	1.13 (1.02-1.25)	.02	395	1.27 (1.10-1.47)	.002
Hemorrhagic stroke	31	1.56 (0.83-2.94)	.17	32	1.17 (0.71-1.91)	.54
Ischemic stroke	1036	1.11 (1.00-1.24)	.04	362	1.28 (1.10-1.49)	.002
Fatty liver	1777	2.01 (1.85-2.19)	<.001	755	1.69 (1.52-1.89)	<.001
Gallstones	839	1.27 (1.13-1.42)	<.001	204	1.25 (1.03-1.53)	.03
Cholecystitis	301	0.93 (0.77-1.12)	.44	96	1.58 (1.16-2.16)	.004
Gout	119	1.20 (0.88-1.63)	.25	134	1.35 (1.05-1.73)	.02

Abbreviations: HR, hazard ratio; NA, not applicable.

^a^ Healthy weight individuals were those who had a body mass index (calculated as weight in kilograms divided by square of height in meters) of 18.5 to 22.9 at middle adulthood.

^b^ Female ever smokers were excluded from analyses.

^c^ The HRs were adjusted for age at study enrollment, recalled weight at 20 years of age, height, educational level, smoking status, pack-years of smoking, regular alcohol consumption, total physical activity, healthy eating index, family cancer history, menopausal status (women only), female hormone replacement therapy (women only), parity (women only), and birth cohorts.

^d^ Obesity-related cancers included cancers of gastric cardia, colon or rectum, liver, gallbladder, pancreas, renal cell, thyroid, and multiple myeloma.

The Figure (A-D) shows the cumulative risk of death from any cause or CVD among lifetime never smokers by the weight gain of people who reached a BMI of 23 or higher at middle adulthood. Approximately 10.0% of women who gained 20 kg or more would die by 70 years of age compared with a death rate of 6.8% among healthy-weight women. For CVD-related mortality, the rate was 2.9% for women who gained 20 kg or more and 1.2% for healthy-weight women. Similar associations were found among men who had never smoked. Plots of cumulative risk including among ever smokers are shown in eFigure 2 in the Supplement.

**Figure.**
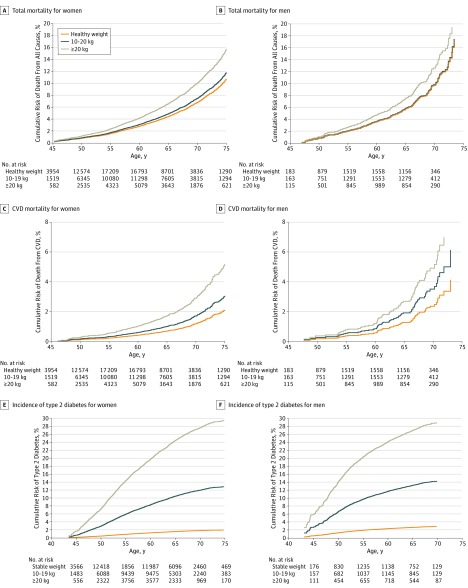
Adjusted Cumulative Mortality for All-Cause and Cardiovascular Diseases (CVDs) Among Lifetime Never Smokers and Cumulative Risk for Type 2 Diabetes Among Women and Men A-D, Healthy weight, a body mass index (BMI, calculated as weight in kilograms divided by height in meters squared) of 18.5-22.9 at middle adulthood; 10-19 kg, gained 10-19 kg leading to BMI ≥23 at middle adulthood; ≥20 kg, gained 20 kg or more leading to BMI ≥23 at middle adulthood. E and F, Stable weight, with a weight change of ±2.5 kg; 10-19 kg, with a weight gain of 10-19 kg; and ≥20 kg, with a weight gain ≥20 kg. Risks were adjusted for enrollment age, recalled weight at age 20 years, height, education, regular alcohol consumption, total physical activity, healthy eating index, family cancer history, and birth cohort; for men only, type 2 diabetes risk was adjusted for smoking status and pack-years; and for women only, all risks were adjusted for menopausal status, hormone replacement therapy, and parity.

Compared with all-cause and CVD mortality, a larger difference in cumulative risks of developing type 2 diabetes was found between those who gained 20 kg or more (29.0% for men and 27.6% for women by 70 years of age) and those with a stable weight (2.9% for men and 1.8% for women). Those who gained a moderate weight of 10.0 to 19.9 kg from early to middle adulthood were also at a considerably elevated risk of developing type 2 diabetes (14.3% for men and 11.9% for women) (Figure, E and F).

## Discussion

In 2 large prospective Chinese cohorts, we found that overall weight gain from early to middle adulthood was associated with multiple disease incidence and mortality outcomes in later life for people who reached a BMI of 23 or higher at middle adulthood. For people who had a BMI of 18.5 to 22.9 at middle adulthood, weight gain was associated with elevated risk of type 2 diabetes, hypertension, ischemic stroke, fatty liver disease, and gallstones.

We found that BMI at middle adulthood modified the association of weight gain from early to middle adulthood with multiple cancer incidence and mortality outcomes. For example, for participants who reached a BMI of 23 or higher at middle adulthood, weight gain was associated with increased risk of overall mortality or CVD mortality in both men and women and cancer mortality in women, whereas for those whose BMI remained in the healthy range (BMI of 18.5-22.9), weight gain was associated with a reduced risk of death (for total, cancer, and other-cause mortality in men) or no excess risk of death. Because a considerable proportion of Asian individuals are lean in early and middle adulthood, the results of our study suggest that some moderate weight gain from early to middle adulthood may be unharmful or even beneficial for those with a low body weight early in life in reducing overall mortality in later life.

In addition to total and CVD-related mortality, we found that overall weight gain was associated with an increased risk of obesity-related cancers in both men and women and postmenopausal breast cancer, liver cancer, and cancer of corpus uteri in women. These findings are, in general, supported by the recent study^11^ conducted in the European ancestry population. However, for those who had a healthy weight at middle adulthood, no elevated risk of cancer was found in association with their weight gain. Of interest, an elevated risk of type 2 diabetes, hypertension, ischemic stroke, fatty liver disease, and gallstones was associated with weight gain in both men and women even among those who had a healthy BMI of 18.5 to 22.9 at middle adulthood after the weight gain. For men, an association was also found for increased risk of acute myocardial infarction, cholecystitis, and gout. These findings perhaps could be explained by the higher susceptibility to insulin resistance of Asian individuals than European individuals.^13,17^ Adverse effects of excess adiposity include overproduction of hormones and adipokines, chronic inflammation, and insulin resistance.^25,26^ It is possible that some Chinese adults might experience some insulin resistance even at a BMI of 18.5 to 22.9 at middle adulthood. Further research is needed to identify reasons for the elevated risk of these chronic diseases among Chinese adults with a healthy weight at middle adulthood.

To our knowledge, this is the largest prospective cohort study that systematically examined the association of weight gain from early to middle adulthood with multiple health outcomes. A large fraction of the study participants had a normal BMI at middle adulthood, providing us a unique opportunity to investigate a potential modifying effect of BMI at middle adulthood on the association between weight gain and disease outcomes.

### Limitations

This study has several limitations. One limitation is that weight at early adulthood was self-reported retrospectively at the baseline survey in middle adulthood, which may have some recall errors. The true associations may have been underestimated if the recall error is nondifferential. In addition, there could be a survival bias in this study because study participants had to live until middle adulthood to be recruited in our study. However, the mortality from early to middle adulthood was low for residents in Shanghai (an estimated rate of approximate ≤1% from 20-50 years of age).^27^ Furthermore, we excluded individuals with BMIs less than 18.5 at 20 years of age. Therefore, the potential influence of survival bias on our results should be small. Another limitation is that some residual confounding from tobacco smoking may exist, although we have adjusted for tobacco smoking carefully. Smokers usually have lower weight gain than never smokers, and the confounding effects of smoking tend to attenuate the association between weight gain and health outcomes.^28^ Nevertheless, we found a similar pattern of associations among never smokers, indicating that potential residual confounding from tobacco smoking should not be substantial. Our study included a large number of lifetime never-smoker women, enabling a rigorous investigation free of potential confounding from tobacco smoking.

## Conclusions

Weight gain from early to middle adulthood may be associated with the incidence and mortality of multiple diseases in later life, and some of these associations may be modified by BMI at middle adulthood. Our study highlights the importance of maintaining a healthy weight throughout life.

## Notes

Supplement. **eTable 1.** Selected Baseline Characteristics of Study Participants in the Shanghai Women’s Health Study and Shanghai Men’s Health Study (1996-2006) **eFigure 1.** Dose-Response Association Between Weight Gain From Early to Middle Adulthood and Mortality for All-Cause and CVDs Among Subjects Who Had a BMI ≥23 at Middle Adulthood **eTable 2.** Adjusted Hazard Ratios for Total and Cause-Specific Mortality Per 5 kg Weight Gain From Early to Middle Adulthood Stratified by Smoking Status, SMHS, 2002-2014 **eFigure 2.** Adjusted Cumulative Mortality for All-Cause and CVDs Among Men
