# Supplementary material for: Association of Adult Weight Gain With Major Health Outcomes Among Middle-aged Chinese Persons With Low Body Weight in Early Adulthood
Source: JAMA Netw Open. 2019 Dec 13;2(12):e1917371. doi: 10.1001/jamanetworkopen.2019.17371 (PMC6991199; doi:10.1001/jamanetworkopen.2019.17371)
Supplement: Supplement. — eTable 1. Selected Baseline Characteristics of Study Participants in the Shanghai Women’s Health Study and Shanghai Men’s Health Study (1996-2006) eFigure 1. Dose-Response Association Between Weight Gain From Early to Middle Adulthood and Mortality for All-Cause and CVDs Among Subjects Who Had a BMI ≥23 at Middle Adulthood eTable 2. Adjusted Hazard Ratios for Total and Cause-Specific Mortality Per 5 kg Weight Gain From Early to Middle Adulthood Stratified by Smoking Status, SMHS, 2002-2014 eFigure 2. Adjusted Cumulative Mortality for All-Cause and CVDs Among Men [file jamanetwopen-2-e1917371-s001.pdf]

Supplementary Online Content

Jia G, Shu X-O, Liu Y, et al. Association of adult weight gain with major health outcomes among middle-aged Chinese persons with low body weight in early adulthood. *JAMA Netw Open*. 2019;2(12):e1917371. doi:10.1001/jamanetworkopen.2019.17371

eTable 1. Selected Baseline Characteristics of Study Participants in the Shanghai Women's Health Study and Shanghai Men's Health Study (1996-2006)

eFigure 1. Dose-Response Association Between Weight Gain From Early to Middle Adulthood and Mortality for All-Cause and CVDs Among Subjects Who Had a BMI ≥23 at Middle Adulthood

eTable 2. Adjusted Hazard Ratios for Total and Cause-Specific Mortality Per 5 kg Weight Gain From Early to Middle Adulthood Stratified by Smoking Status, SMHS, 2002-2014

eFigure 2. Adjusted Cumulative Mortality for All-Cause and CVDs Among Men

This supplementary material has been provided by the authors to give readers additional information about their work.

© 2019 Jia G et al. *JAMA Network Open*.

eTable 1. Selected Baseline Characteristics of Study Participants in the Shanghai Women's Health Study and Shanghai Men's Health Study (1996-2006)

**Characteristics**	**Women** (n = 48,377)	**Men** (n = 35,989)
**Age, year, mean (SD)**	47.8 (5.3)	49.6 (5.1)
**Height, cm, mean (SD)**	158.7 (5.2)	171.0 (5.6)
**Body weight at age 20, kg, mean (SD)**	49.7 (6.5)	57.1 (6.4)
**BMI at baseline, mean (SD)**	23.8 (3.1)	23.9 (2.8)
BMI ≥ 23, n (%)	27,223 (56.3)	21,771 (60.5)
**Weight gain, kg, mean (SD)**	10.3 (8.6)	12.8 (9.1)
Weight loss > 2.5 kg, n (%)	3,277 (6.8)	1,465 (4.1)
Weight loss ≤ 2.5 kg or weight gain < 2.5 kg, n (%)	4,999 (10.3)	2,9191 (8.1)
Weight gain ≥ 2.5 kg and < 10.0 kg, n (%)	14,587 (30.2)	8,894 (24.7)
Weight gain ≥ 10.0kg and < 20.0 kg, n (%)	19,198 (39.7)	15,102 (42.0)
Weight gain ≥ 20.0kg, n (%)	6,316 (13.1)	7,609 (21.1)
**Education, n (%)**		
Elementary or middle school	24,294 (50.2)	8,518 (37.6)
High school graduate	16,559 (34.2)	12,954 (41.6)
Some college or more	7,524 (15.6)	7,490 (20.8)
**Total physical activities, MET-hr/wk per year, median (IQR)**	99.23 (73.8, 131.3)	53.8 (30.8, 70.7)
**Smoking status, n (%)**		
Never smoker	48,377 (100)	8,518 (23.7)
Ever smoker	0	27,471 (76.3)
**Pack-years of smoking, mean (SD)** [a]		17.2 (15.4)
**Healthy eating index, mean (SD)** [b]	44.6 (7.8)	42.6 (8.3)
**Family history of cancer, n (%)**	13,417 (27.7)	10,495 (29.2)
**Ever diagnosed at baseline, n (%)** [c]		
Coronary heart disease	1,784 (3.7)	451 (1.3)
Diabetes mellitus	1,104 (2.3)	1,388 (3.9)
Hypertension	8,441 (17.4)	7,853 (21.8)
Chronic Obstructive Pulmonary Disease	2,857 (5.9)	747 (2.1)
Chronic gastritis	9,184 (19.0)	5,196 (14.4)
**Postmenopausal, n (%)**	14,445 (29.9)	
**Ever used female hormone replacement therapy, n (%)**	2,102 (4.3)	
**Parity, n (%)**		
1 Child	35,521 (73.4)	
>=2 Children	11,348 (23.5)	

Abbreviation: IQR, interquartile range; SD, standard deviation.
[a] Ever-smoking women were excluded from analyses.
[b] Alternative healthy eating index was developed from the dietary guidelines for Americans and modified by Harvard researchers. Details of the index have been described in previous publications [20].
[c] Ever diagnosed with the disease prior to the baseline survey.

© 2019 Jia G et al. *JAMA Network Open*.

a). Total mortality of women

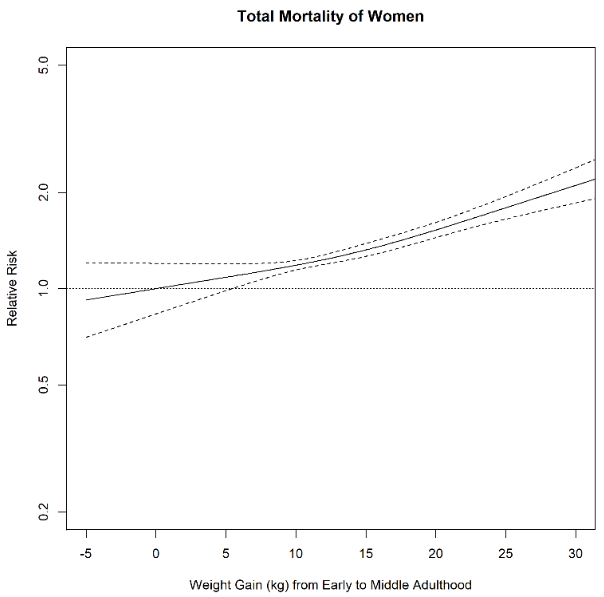

b). CVD mortality of women

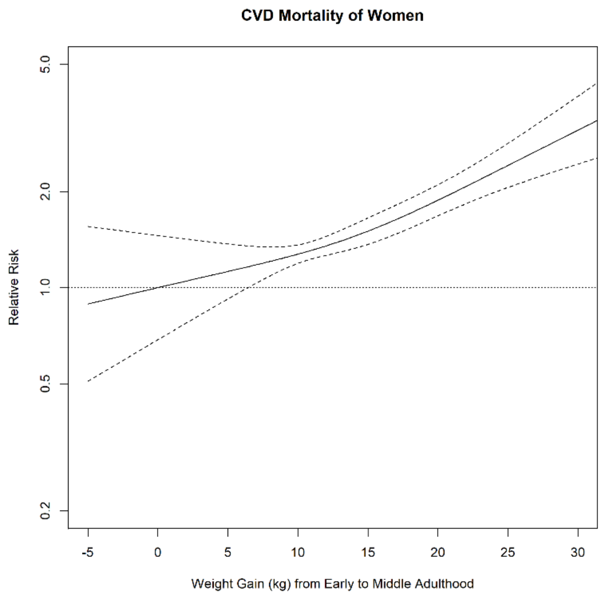

c). Total mortality of men

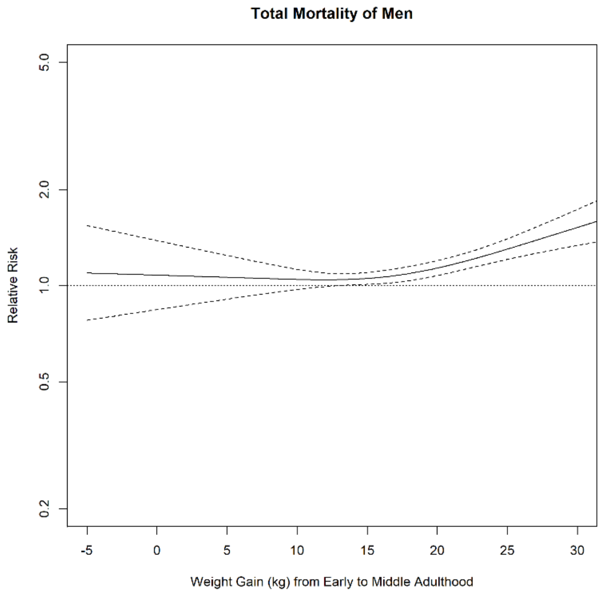

d). CVD mortality of men

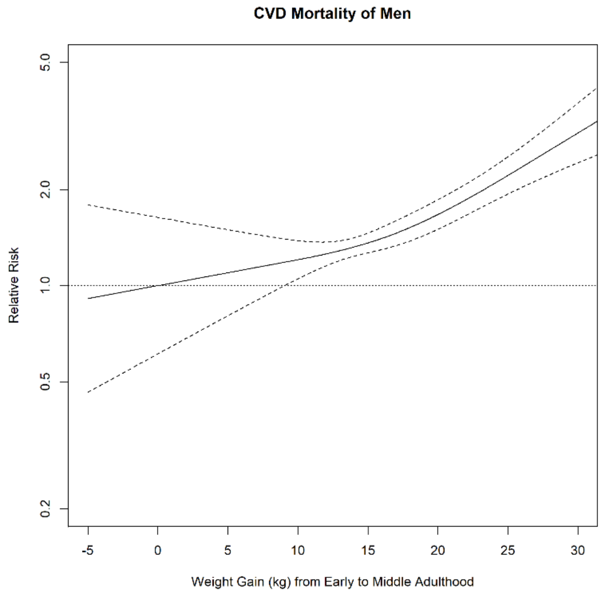

eFigure 1. Dose-response association between weight gain from early to middle adulthood and mortality for all-cause and CVDs among subjects who had a BMI ≥ 23 at middle adulthood. Spline regression was conducted with 3 knots and 0 kg weight change as reference; Female ever-smokers were excluded from analyses; Hazard ratios were adjusted for age at study enrollment, recalled weight at age 20, height, educational level, smoking status, pack-years of smoking, regular alcohol consumption, total physical activity, alternative healthy eating index (AHEI), family cancer history, menopausal status (women only), female hormone replacement therapy (women only), parity (women only), chronic diseases (type 2 diabetes, hypertension, COPD, coronary heart disease, and chronic gastritis) at baseline, and birth-cohorts.

© 2019 Jia G et al. *JAMA Network Open*.

eTable 2. Adjusted hazard ratios for total and cause-specific mortality per 5 kg weight gain from early to middle adulthood stratified by smoking status, SMHS, 2002-2014

Mortality	Total			18.5 < BMI < 23			BMI ≥ 23			*P* for interaction
	No. of deaths	HRs (95% CI) [a]	*P* value	No. of deaths	HRs (95% CI) [a]	*P* value	No. of deaths	HRs (95% CI) [a]	*P* value	
**Male never-smokers**	n = 8,518			n = 2,978			n = 5,540			
All-cause deaths	313	1.06 (0.98, 1.14)	0.135	94	0.82 (0.62, 1.10)	0.191	219	1.07 (0.95, 1.19)	0.268	0.737
CVD deaths	73	1.23 (1.06, 1.43)	0.006	18	0.64 (0.34, 1.23)	0.184	55	1.29 (1.06, 1.57)	0.012	0.479
Cancer deaths	163	1.01 (0.91, 1.12)	0.820	51	1.00 (0.67, 1.51)	0.991	112	0.96 (0.81, 1.13)	0.601	0.779
Other deaths	77	1.00 (0.86, 1.16)	0.957	25	0.66 (0.38, 1.15)	0.143	52	1.05 (0.83, 1.33)	0.665	0.732
**Male ever-smokers**	n= 27,471			n = 11,240			n = 16,231			
All-cause deaths	1,331	0.99 (0.95, 1.02)	0.357	569	0.83 (0.74, 0.93)	0.001	762	1.10 (1.04, 1.16)	0.001	0.034
CVD deaths	326	1.12 (1.05, 1.19)	0.001	124	0.99 (0.77, 1.26)	0.914	202	1.26 (1.15, 1.38)	<0.001	0.056
Cancer deaths	724	0.94 (0.90, 0.99)	0.009	325	0.78 (0.67, 0.91)	0.001	399	1.04 (0.96, 1.13)	0.307	0.518
Other deaths	281	0.94 (0.88, 1.01)	0.107	120	0.82 (0.64, 1.06)	0.126	161	1.01 (0.89, 1.14)	0.896	0.272

Abbreviation: BMI, body mass index; CI, confidence interval; HR, hazard ratio.

[a] HRs were adjusted for age at study enrollment, recalled weight at age 20, height, educational level, smoking status (ever-smokers only), pack-years of smoking (ever-smokers only), regular alcohol consumption, total physical activity, healthy eating index, family cancer history, and birth-cohorts.

© 2019 Jia G et al. *JAMA Network Open*.

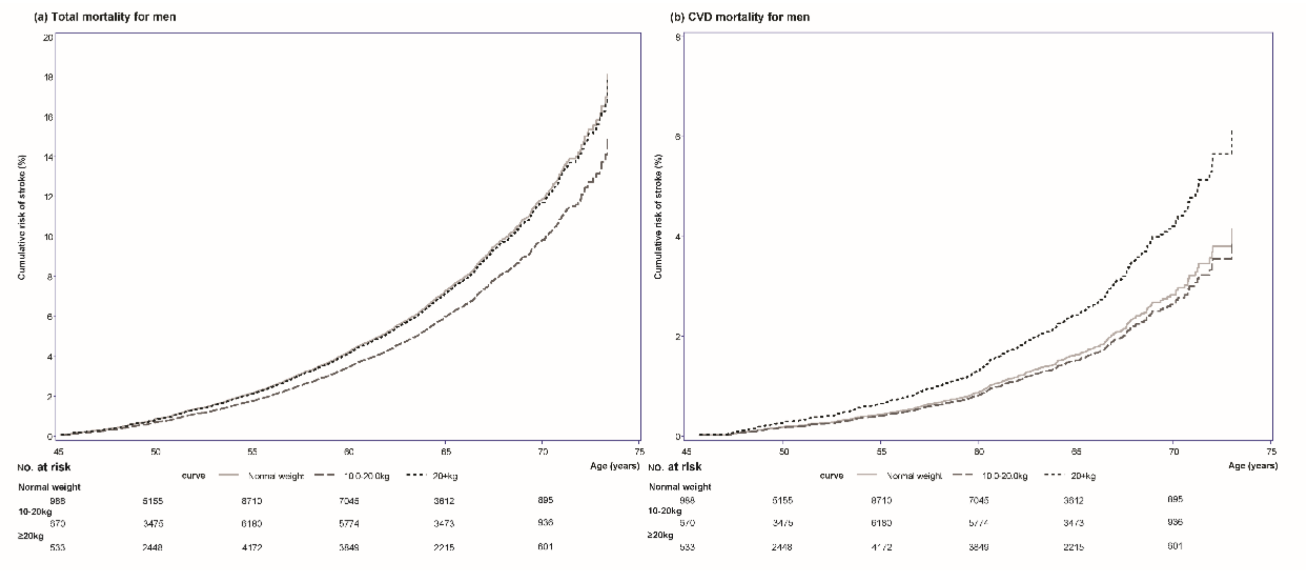

eFigure 2. Adjusted cumulative mortality for all-cause and CVDs among men. “Normal weight” group refers to participants who had a BMI in 18.5–22.9 at middle adulthood. “10–20kg” and “20+kg” groups refer to participants who gained 10–20 kg or more than 20 kg body weight from early to middle adulthood and reached a BMI ≥ 23 at middle adulthood, respectively. Rates were adjusted for age at study enrollment, recalled weight at age 20, height, educational level, regular alcohol consumption, total physical activity, healthy eating index, family cancer history, smoking status, pack-years of smoking, and birth-cohorts.

© 2019 Jia G et al. *JAMA Network Open*.
